# Development of dental implant drill with detection mechanism of bone quality change

**DOI:** 10.1186/s40729-025-00650-6

**Published:** 2025-10-21

**Authors:** Takahiro Nozaki, Seiji Asoda, Soya Shimizu, Ryo Kondo, Koji Niwa, Masaki Yazawa, Kazuo Kishi, Hiromasa Kawana

**Affiliations:** 1 Department of System Design Engineering, Faculty of Science and Technology, Keio University, Yokohama, Kanagawa 223-8522 Japan; 2 Department of Dentistry and Oral Surgery, School of Medicine, Keio University, Shinjuku-ku, Tokyo 160-8582 Japan; 3 Graduate School of Integrated Design Engineering, Keio University, Yokohama, Kanagawa 223-8522 Japan; 4 Department of Plastic and Reconstructive Surgery, School of Medicine, Keio University, Shinjuku-ku, Tokyo 160-8582 Japan; 5 Department of Oral and Maxillofacial Implantology, School of Dentistry, Kanagawa Dental University, Yokosuka, Kanagawa 238-8580 Japan

**Keywords:** Dental implant drill, Robotics, Robotic surgery, Automatic stopping

## Abstract

**Purpose:**

This study aims to develop and evaluate a dental implant drill system capable of preventing maxillary sinus membrane perforation, a common complication in cases with limited alveolar bone height, particularly in the maxillary molar region. The primary objective is to design a mechanism that autonomously detects changes in bone quality and halts drill rotation upon reaching the sinus floor.

**Methods:**

A novel dental implant drill incorporating an integrated bone quality detection mechanism was developed. The system includes a centrally mounted detector that actuates a switch controlling drill rotation. When cortical bone is penetrated and softer tissue is encountered, the detector extends outward, interrupting power to the motor. A penetration test was conducted using a 5 mm thick wooden board as a surrogate bone model to evaluate the drill’s response to cortical penetration.

**Results:**

Experimental trials demonstrated that the drill automatically ceased rotation upon advancing approximately 0.47 mm beyond a simulated bone surface. Given that the maxillary sinus membrane is typically less than 1 mm in thickness, this minimal protrusion indicates a significantly reduced risk of perforation.

**Conclusion:**

The proposed drill system effectively detects transitions in bone quality and prevents over-penetration, offering a promising solution for enhancing surgical safety during maxillary implant procedures.

## Introduction

The integration of robotic technology into medical procedures, including skull base perforation and apicoectomy, is rapidly advancing, as reflected in ongoing research and the development of various surgical assistance and navigation systems [1–4]. In particular, procedures involving bone manipulation demand precise control of drill positioning, rendering robotic assistance especially advantageous for such applications [5–7]. For instance, in spinal surgery, where partial bone removal is required to relieve spinal cord compression, a handheld bone-cutting system has been developed. This system determines the occurrence of cutting by monitoring motor current and drill rotation speed, while an accelerometer detects the contact state at the tool tip [8, 9]. Further studies have focused on deformable structures such as bone, which may bend during cutting. In such cases, control methods have been proposed that utilize high-speed Fourier transformation of the response from a triaxial accelerometer to vibration signals in order to regulate cutting depth and angle. Other approaches estimate the cutting depth between cancellous and cortical bone by analyzing harmonic components in the accelerometer signals [10–12].

The integration of robotics and control engineering has advanced procedures involving bone manipulation. However, these approaches typically require preprocessing steps such as data-driven learning and calibration using artificial bone plates, necessitating prior knowledge of the target object’s geometry and material properties [13, 14]. Despite progress in preoperative imaging techniques, accurately replicating intraoperative conditions remains challenging. Variations in bone density, anatomical structure, and patient positioning often result in inconsistencies between preoperative planning and the actual surgical environment. Consequently, surgeons must frequently rely on their experience and tactile feedback to guide the drilling process, which increases the risk of unintended perforations and incomplete implant placement. Such discrepancies are particularly evident in dental implant procedures. Although dental implants provide substantial benefits-including preservation of natural dentition, improved masticatory function, and enhanced aesthetics-they also carry notable risks. Complications such as damage to the maxillary sinus mucosa are especially prevalent in cases with reduced alveolar bone height in the maxillary molar region. These errors can lead to a range of adverse outcomes, from minor issues such as postoperative pain and swelling to severe complications including nerve injury, sinusitis, and implant failure. These findings not only affect patient well-being but may also necessitate additional surgical interventions [15–19]. These challenges highlight the urgent need for technological advancements that improve the precision and reliability of implant placement.

Implant surgery necessitates meticulous drilling to avert the risk of damaging critical anatomical structures. This has given rise to the development of various safety-assistive technologies aimed at mitigating the risk of perforation. A number of studies have previously been conducted in an attempt to enhance the safety of surgical procedures through the use of optical and electrical sensing techniques. These include multispectral optical detection, the purpose of which is to discriminate between bone and soft tissue interfaces [20, 21], and impedance-sensing drills which are designed to alert surgeons before reaching critical structures [22–24]. However, these approaches are encumbered by significant limitations: optical systems are frequently compromised by blood and debris, which attenuate or scatter signals, and they also require external devices that complicate sterilisation and handling. Impedance-based methods are highly sensitive to bone density and hydration state, and thus precise electrode placement and conductive conditions are required, which reduces their reliability and clinical applicability.

In order to address these limitations, this study proposes a novel dental implant drill incorporating a bone quality detection mechanism that integrates a rounded detection pin and mechanical switch directly into the drill tip. The design of the apparatus is intended to minimise the influence of blood and hydration state, and no additional external equipment is required. It enables immediate mechanical stoppage of the drill, thus offering a simple and clinically applicable safety mechanism. The system has been designed to enhance the precision, safety, and predictability of implant placement by reducing dependence on the surgeon’s subjective assessment. The result is a minimisation of the risk of complications and an improvement in long-term treatment outcomes.

This paper presents the design and development of the proposed bone quality detection system, followed by an analysis of its potential advantages and limitations. The final section summarizes the key findings and outlines directions for future research.

**Fig. 1:**
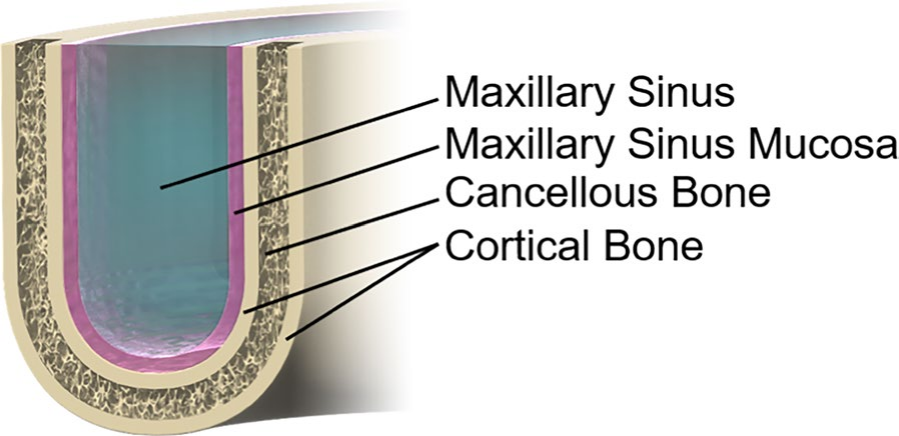
Structure of Maxilla

## Necessity of penetration detection in dental implants

**Fig. 2:**
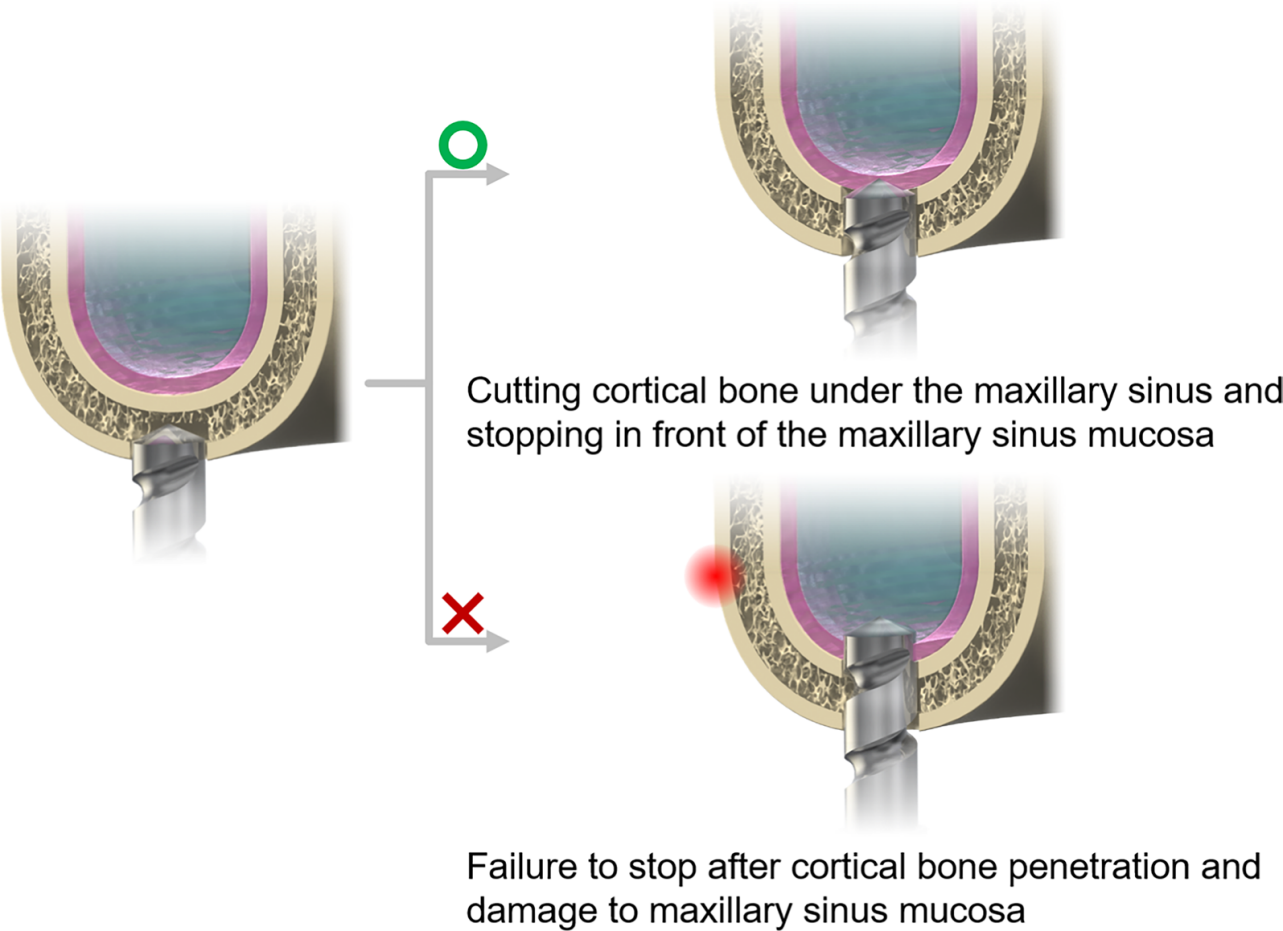
Damage to the maxillary sinus mucosa caused by the drill following penetration of the cortical bone

The nasal cavity is surrounded by several anatomical spaces collectively referred to as the paranasal sinuses. Among them, the maxillary sinus-located in the cheek region-is the largest. These sinuses perform several important functions, including shock absorption during facial trauma, nasal mucus secretion, and contribution to vocal resonance. The maxillary sinus is lined by the Schneiderian membrane (also referred to as the maxillary sinus mucosa), which serves as a critical barrier that protects against the intrusion of foreign substances. The maxillary region has a stratified anatomical structure (Fig. 1), progressing from the outer surface inward as follows: high-density cortical bone, lower-density cancellous bone, another layer of cortical bone, the maxillary sinus mucosa, and finally the sinus cavity. In healthy individuals, the thickness of the mucosal layer is less than 1 mm, with no correlation to age or sex [25–28]. It is important to note that the sinus membrane possesses inherent elasticity, and clinical reports from sinus lift procedures demonstrate that it can withstand deformation of several millimeters without rupture [29]. The sinus membrane’s elastic property offers a potential safety margin in the event of immediate cessation of drilling upon contact, thereby enabling the tissue to deform slightly without perforation. However, in the event of perforation of the membrane, serious complications can ensue, as demonstrated in Fig. 2. It is imperative to detect structural penetration into the maxillary region with precision during surgical procedures to avoid damaging critical anatomical components. Thus, immediate detection and cessation of drilling are imperative in order to ensure patient safety.

In dental implant procedures, precise control of drilling depth is critical when approaching the cortical bone underlying the maxillary sinus. The drilling must be terminated before breaching the maxillary sinus mucosa, as failure to detect the transition from bone to soft tissue can result in mucosal perforation, potentially leading to complications such as maxillary sinusitis [30–32]. This challenge is particularly pronounced when the operator is unable to respond appropriately to the abrupt decrease in resistance that occurs upon penetrating the cortical bone, thereby increasing the risk of unintended intrusion into the sinus mucosa.

Although surgical guides are commonly used to reduce this risk, they are associated with several limitations, including spatial discrepancies between preoperative planning parameters and actual intraoperative positioning. Additionaly, the fabrication of surgical guides imposes financial and time-related burdens on patients.

To address these challenges, this study introduces a novel dental implant drill incorporating a bone quality change detection mechanism. This system is designed to enable appropriate treatment while preventing perforation of the maxillary sinus mucosa, independent of the operator’s level of expertise or the patient’s anatomical variations. The following section provides a detailed description of the drill’s design.

**Fig. 3:**
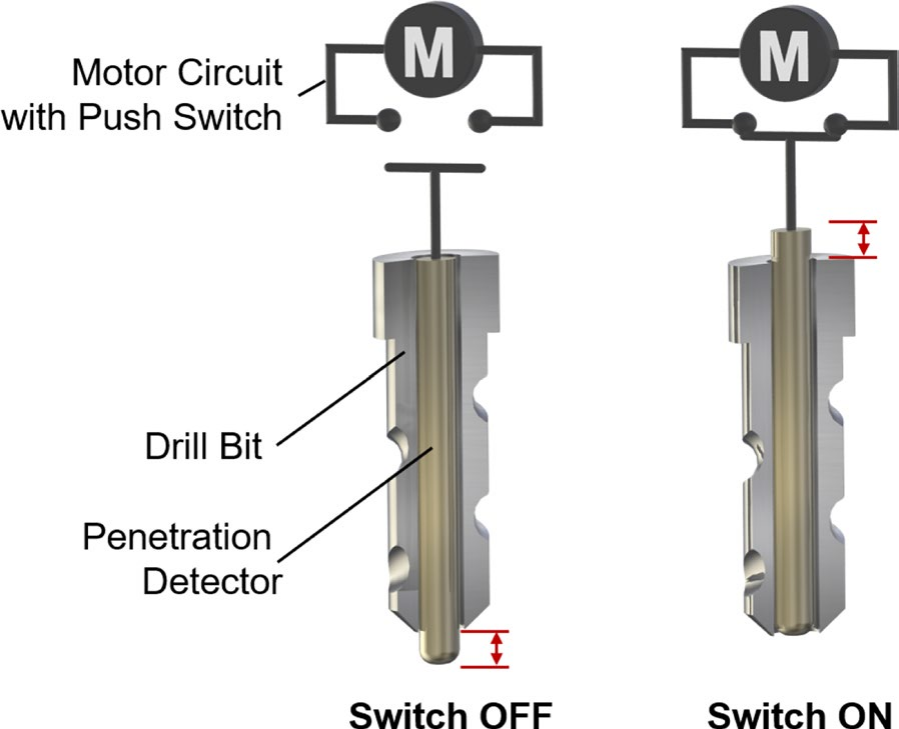
Schematic of the dental drill incorporating a bone quality detection mechanism. The detector is centrally located within the drill and connected to a push-button switch integrated into the motor circuit. Left: When the detector protrudes beyond the drill tip, the circuit remains open, and the drill does not rotate. Right: When the detector is pressed inward upon contact with the target material, the circuit closes, enabling drill rotation

**Fig. 4:**
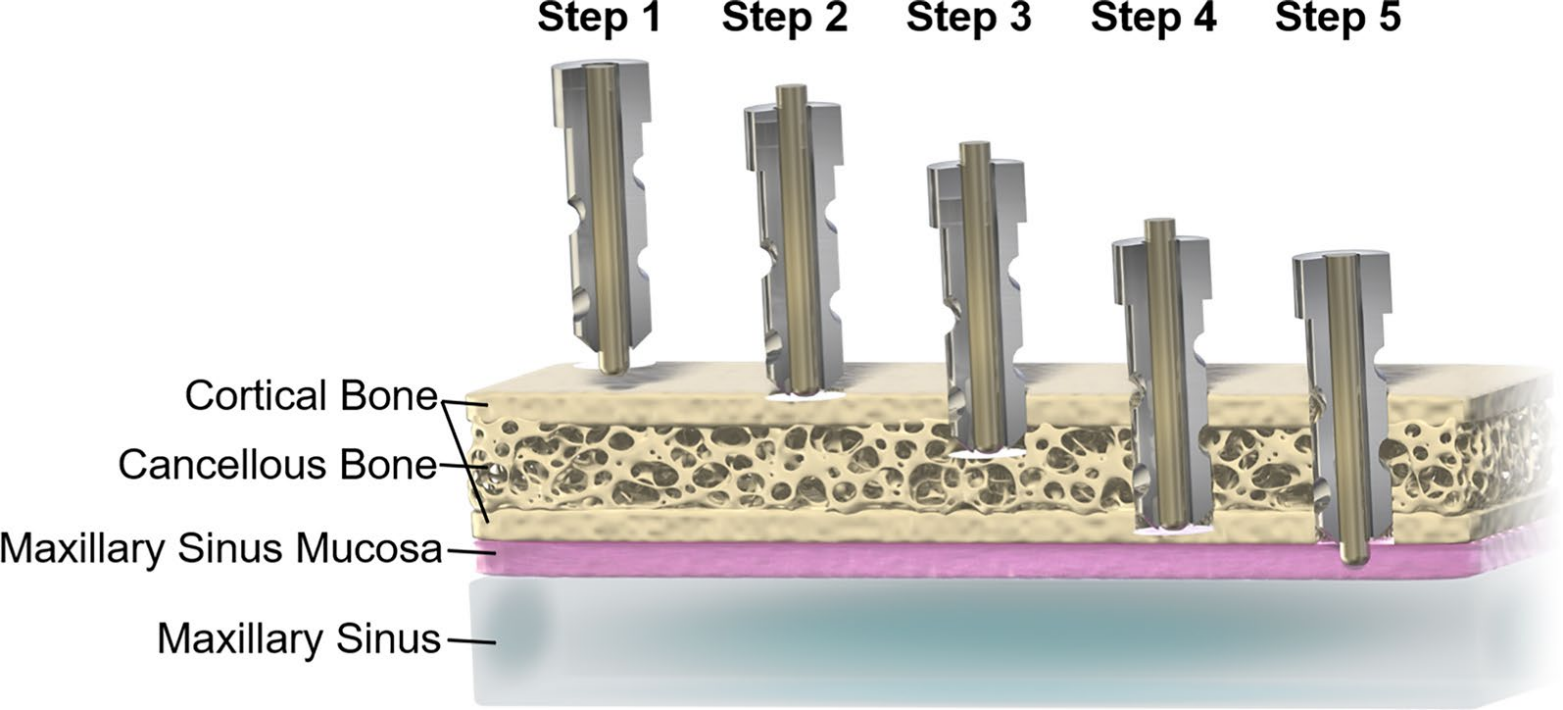
Procedural depiction of maxillary surgery using the drill with bone quality detection: Step 1: The drill is positioned near the target; the detector protrudes, and the drill remains stationary. Step 2: Upon contact with the outer cortical bone, the detector is depressed, and drilling begins. Step 3: The drill advances through cancellous bone; although softer than cortical bone, it maintains sufficient resistance to keep the detector depressed, allowing drilling to continue. Step 4: The drill reaches the inner cortical bone. Step 5: Upon contact with the sinus membrane, the detector extends outward, opening the circuit and stopping rotation, thereby preventing perforation

## Mechanism of drill and implant method

This section presents a detailed description of the proposed dental implant drill, which is equipped with a bone quality change detection mechanism.

### Mechanism of drill

As illustrated in Fig. 3, the drill comprises two primary components: a conventional drill bit and a detector integrated coaxially within the drill bit’s hollow core. The detector is engineered to move independently along the central axis, thereby enabling real-time assessment of tissue resistance during drilling. Of particular note is the detection mechanism, which incorporates a rounded, non-cutting tip that protrudes once the cortical analogue becomes thin, thereby ensuring that the sinus membrane is not directly subjected to cutting force.

A mechanical switch, coupled with a spring, is installed at the proximal end of the detector to form a safety interlock mechanism. In its default configuration, the detector protrudes beyond the distal tip of the drill bit, thereby ensuring that the switch remains in an open state and preventing activation of the drill even when power is supplied. The system is designed to initiate rotation exclusively when the detection pin is pressed inward, thereby precluding any possibility of idle rotation outside the oral cavity. This feature is intended to enhance operational safety. Upon contact with bone, the detector retracts axially, thereby closing the switch and completing the electrical circuit. This, in turn, activates the drill, leading to the initiation of bone cutting.

The system functions on the basis of a simple mechanical principle, as opposed to utilising complex pressure-sensing mechanisms. The sensor circuit is mechanically opened by the protrusion of the detection pin, which is caused by a reduction in tissue resistance. This results in the immediate cessation of both forward motion and drill rotation. Upon reaching the sinus membrane, the detection pin protrudes by several millimeters; however, the sinus membrane’s inherent elasticity acts as an additional safety margin, accommodating this pin protrusion without rupture. The system’s inherent elastic property enables the automatic cessation of drill rotation, thereby preventing membrane perforation. It is therefore the case that once the sinus membrane is reached, the cutting action itself is terminated, thereby minimising the risk of membrane damage. The configuration under discussion imposes constraints on the execution of drilling operations, thereby ensuring that such operations are conducted exclusively within parameters in which sufficient resistance is detected. This, in turn, serves to mitigate the risk of unintended tissue penetration.

### Implant method

Figure 4 schematically depicts the drill’s operational sequence during maxillary implant placement. Before contacting the bone, the detector remains in its extended default position, keeping the internal switch deactivated and the drill inoperative. Upon contacting the bone surface, the detector retracts, actuating the switch and enabling drill rotation for bone removal.

As the drill advances through the dense cortical bone and into the less dense cancellous layer, the reduced resistance continues to maintain partial retraction of the detector, allowing uninterrupted drilling. However, upon reaching the soft maxillary sinus mucosa, the detector encounters minimal resistance, allowing it to fully extend. This motion reopens the switch, immediately halting drill rotation and thereby preventing perforation of the sinus membrane.

The integrated on/off switching mechanism enables safe and precise maxillary implant procedures by protecting the maxillary sinus mucosa. The incorporation of this detection system significantly reduces the risk of accidental perforation, providing a more reliable and controlled alternative to conventional drilling techniques. The subsequent section presents the results of experiments conducted to evaluate the practicality of the proposed drill with a penetration detection mechanism.

**Fig. 5:**
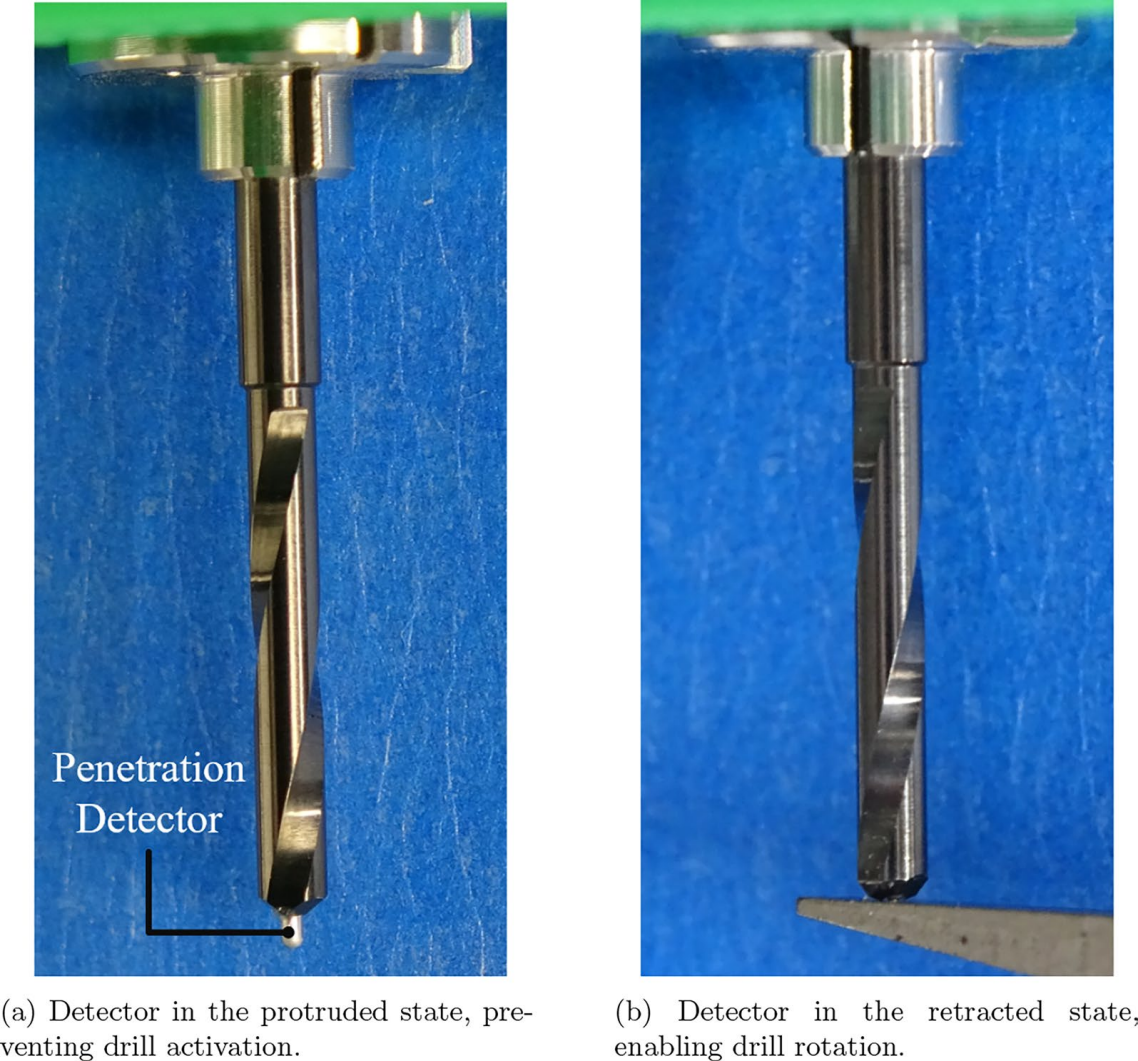
Photograph of the prototype drill equipped with the perforation detection function

**Fig. 6:**
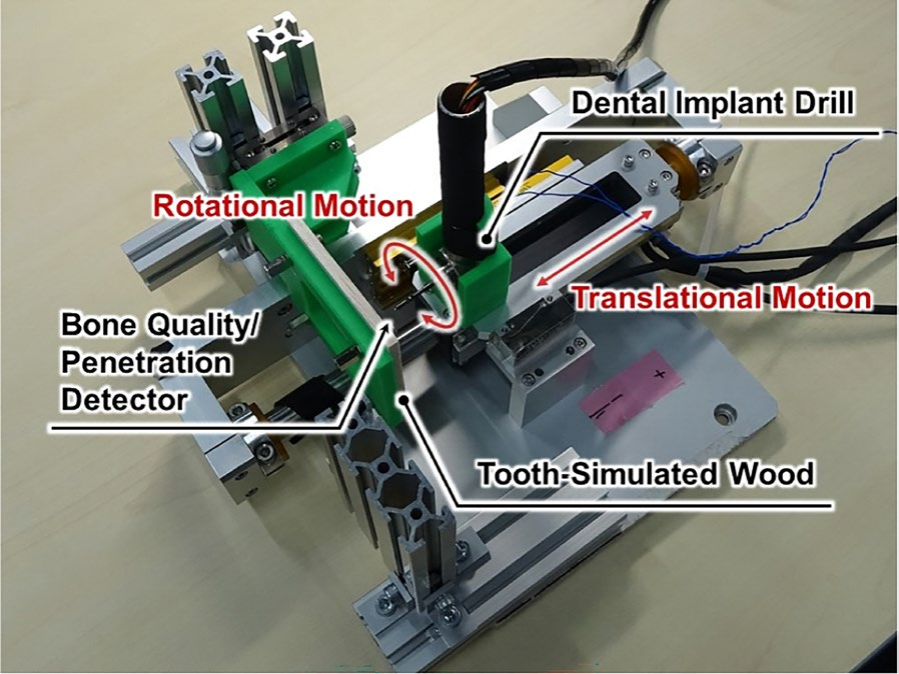
Overview of the experimental apparatus, including the drill with a penetration detection mechanism and tooth simulated wood (surrogate bone). The drill moves vertically relative to the board. Upon penetration, the detection pin protrudes, stopping the drill’s rotation

## Experimental results of drilling with propose drill

This section describes the fabrication of a prototype based on the theoretical design outlined previously and assesses its performance through experimental validation under simulated surgical conditions. A series of experimental methods were employed to empirically confirm the feasibility and technical reliability of the system.

### Experimental environment

The experimental setup, including the actual drill used, is shown in Fig. 5. The drill bit has a diameter of 2 mm, consistent with standard pilot drills used in clinical dental implant procedures. The central detection pin-responsible for identifying changes in bone quality-has a diameter of 0.6 mm and is designed to protrude 0.5 mm beyond the drill tip to ensure accurate detection. The axial movement of the detection pin is regulated by a compression spring with a spring constant of 14.024 N/mm and a maximum load of 18.93 N. This spring mechanism enables the pin to retract when encountering dense bone and to extend when contacting softer tissue. The drill is powered by a Maxon ECXSP13L BL KL A HP 24V motor, which provides precise rotational control essential for surgical applications.

**Fig. 7:**
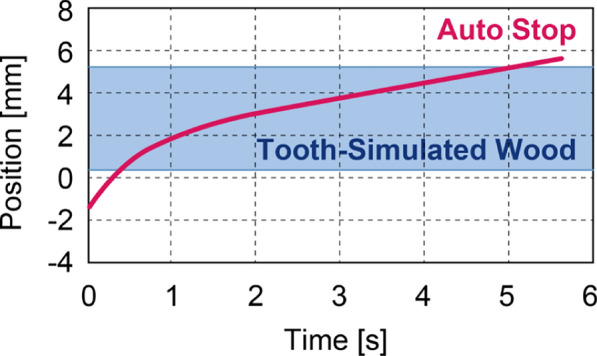
Positional response of the drill. Contact with the simulated bone occurred at 0.35 mm, with the automatic stop triggered at approximately 6 mm

**Fig. 8:**
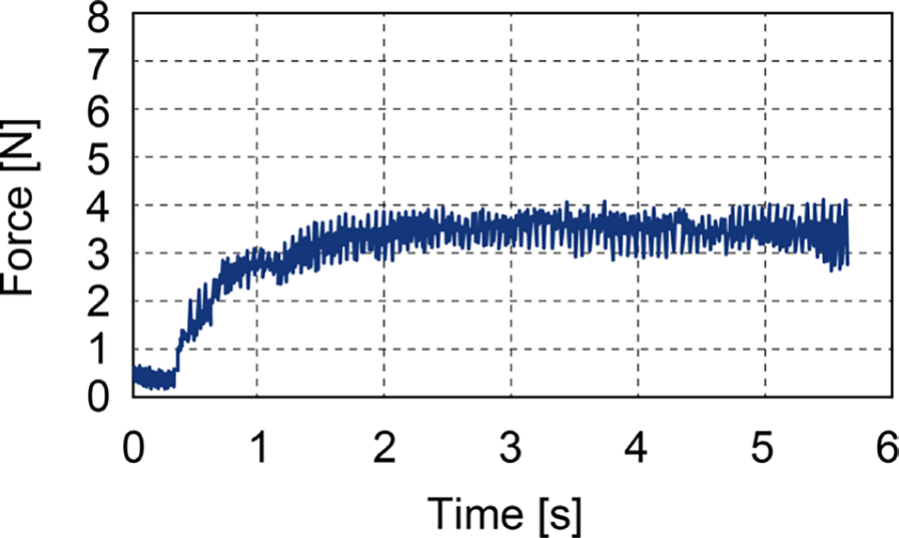
Force response during drilling with a 4 N load. A pressing force of approximately 4 N was maintained after the drill contacted the simulated bone

The experimental setup is illustrated in Fig. 6. In order to evaluate the functionality and reliability of the proposed dental implant drill, a custom-made pilot drill system was designed and tested in bench-top experiments.

A 5-mm-thick veneer board was employed as a bone analogue for the initial validation experiments. Veneer is constituted of a relatively hard surface layer and a softer inner layer, which enables partial reproduction of the difference in hardness between cortical and cancellous bone. According to Misch’s classification, the resistance of veneer corresponds to D2–D3 bone quality, thus making it a reasonable analogue for first-step validation of the detection mechanism. Despite the fact that veneer does not reproduce the porosity, hydration, or anisotropy of human bone, its use permitted a reproducible, low-cost, and controlled evaluation of the device.

The drill system has been engineered in such a manner that rotation commences exclusively upon the application of pressure to the detection pin, thereby ensuring the prevention of unintended activation. This is due to the fact that rotation becomes impossible outside the oral cavity. The drill was mounted on a controlled linear motion system that enabled perpendicular movement relative to the board surface, while continuously recording positional and force data.

The veneer bone surrogate was placed at a depth of 0.35 mm, after which drilling tests were conducted under two controlled loading conditions of 4 N and 5 N, applied in the axial direction. It is important to note that these force values represent cutting load conditions, as opposed to threshold forces. This enables an examination of the behaviour of the detection mechanism under realistic operational parameters. The system was programmed to automatically terminate the drill’s forward and backward motion if a penetration state persisted for more than 0.2 s. Each experimental condition was replicated 12 times. The data were analysed using descriptive statistics and t-tests to evaluate differences between conditions.

### Experimental results

**Fig. 9:**
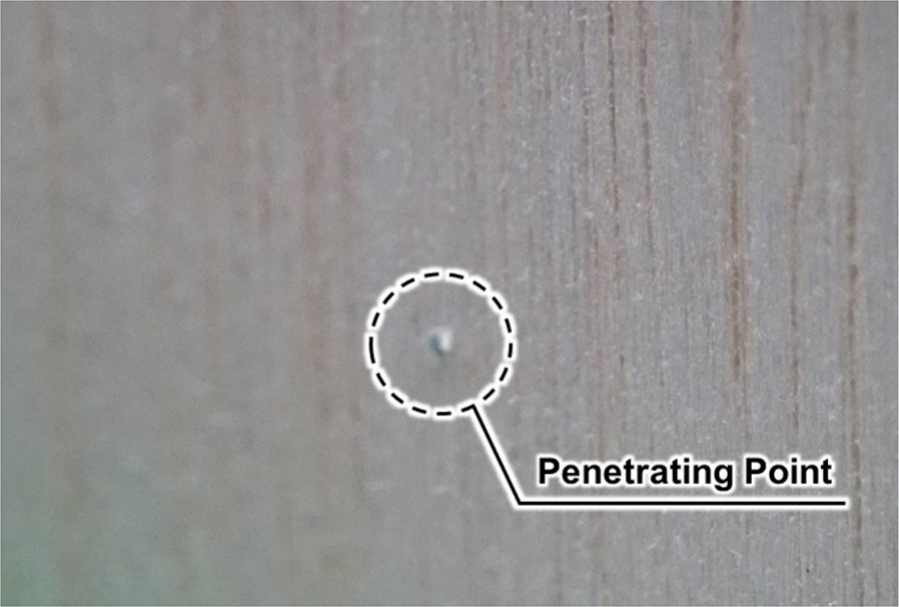
Result of the penetration experiment. The automatic stop mechanism was activated immediately upon drill penetration of the simulated bone

Representative results are presented in Figs. 7 and 8. Figure 7 depicts the positional response of the drill along the linear motion axis, while Fig. 8 displays the corresponding force response. Figure 9 shows the veneer board following automatic drill cessation upon penetration detection, demonstrating the immediate stop after breakthrough.

As outlined in Table 1, a comprehensive statistical analysis was conducted for both 4 N and 5 N loading conditions, as detailed in the table provided. In the 4 N condition, the mean residual bone thickness at the time of sensor activation was recorded as 0.47 mm (95 % CI 0.34$-$0.59). This finding suggests that the process was terminated prior to perforation, a consequence of the vibration-induced stress. This outcome is indicative of a safety-oriented conservative configuration. It is important to note that the typical physiological thickness of the maxillary sinus membrane is less than 1 mm, which provides a favourable safety margin. Conversely, under the 5 N condition, perforation occurred with consistent regularity, exhibiting an average overshoot of 0.85 mm (95 % CI 0.56$-$1.13) and a maximum of 1.7 mm. This setting, oriented towards efficiency, resulted in a substantial augmentation in perforation risk. A t-test was conducted, revealing a statistically significant difference between the two conditions (*p* = 0.027). This finding demonstrates the trade-off between safety and efficiency in the performance of the detection mechanism.

With regard to the reduction of time, the 5 N condition resulted in a statistically significant reduction of approximately 1 s in comparison with the 4 N condition (*p* = 0.012). Furthermore, the drill’s travel speed exhibited an approximate increase of 0.5 mm/s under the 5 N load, which proved to be statistically significant (*p* = $5.52 \times 10^{-5}$). This finding suggests enhanced efficiency and the possibility of reducing the overall duration of the surgical procedure. However, it is important to note that the 4 N condition demonstrated a lower risk of perforation, indicating superior safety performance.

The initial travel distance required for the detection pin to be fully depressed and trigger the drilling operation was approximately 0.2 mm under both loading conditions. No statistically significant difference was observed between the 4 N and 5 N cases (*p* = 0.077 > 0.050). This finding suggests that the activation distance is predominantly determined by the mechanical characteristics of the spring and the initial protrusion length of the detection pin, rather than by the applied cutting load.

The experimental results presented in this section confirm the fundamental effectiveness of the proposed medical drill equipped with a safety mechanism. The findings demonstrate the efficacy of the detection mechanism in reliably distinguishing transitions from harder to softer material, with overshoot values limited to less than 2 mm. The sensor is mounted directly on the drill tip, and it is subjected to constant pressing force during cutting. As a result, mechanical play or backlash in the drive system does not influence detection accuracy. Therefore, the overshoot values reported in this study are indicative of the system’s actual limits. However, the findings also highlight the need for further system optimisation and more comprehensive clinical validation. It is recommended that future research involve testing under a wider range of loading conditions, the use of diverse biological tissue analogues, and evaluation within actual physiological environments.

**Table 1:** Summary of drill performance under cutting loads of 4 N and 5 N

	Protrusion [mm]		Duration [s]		Velocity [mm/s]		Drilling start distance [mm]	
No.	4 N	5 N	4 N	5 N	4 N	5 N	4 N	5 N
1	0.59	0.45	5.06	3.59	1.07	1.47	0.19	0.17
2	0.34	0.19	6.11	3.86	0.83	1.29	0.26	0.20
3	0.34	0.96	4.64	3.94	1.11	1.45	0.19	0.25
4	(− 1.62)	1.71	2.62	5.60	1.24	1.14	0.15	0.32
5	(− 0.68)	0.68	5.09	3.93	0.83	1.38	0.09	0.25
6	0.37	0.70	5.24	3.41	0.99	1.60	0.16	0.24
7	0.35	0.54	5.01	3.40	1.06	1.56	0.02	0.25
8	0.83	0.50	4.82	3.54	1.13	1.47	0.36	0.29
9	0.35	1.58	4.41	3.03	1.14	2.11	0.32	0.17
10	(− 0.82)	0.89	3.19	3.36	1.27	1.67	0.14	0.26
11	0.57	0.80	4.1	2.50	1.30	2.23	0.23	0.22
12	0.45	1.19	4.23	2.77	1.25	2.12	0.17	0.31
Average + SD	0.47 ± 0.17	0.85 ± 0.45	4.54 ± 0.94	3.58 ± 0.78	1.10 ± 0.16	1.63 ± 0.35	0.19 ± 0.09	0.25 ± 0.05
95 % CI	0.34–0.59	0.56–1.13	3.95–5.14	3.08–4.07	1.00–1.20	1.40–1.85	0.13–0.25	0.21–0.28
*p* value	0.027		0.012		$5.52 \times 10^{-5}$		0.077	

The table reports the protrusion length beyond the surrogate bone (Protrusion), cutting duration (Duration), drill movement velocity (Velocity), and advancement distance prior to cutting initiation when the detection pin is engaged (Drilling start distance). Average values, standard deviations (SD), 95 % confidence intervals (CI), and *p* values from t-tests are provided for each parameter. For the 4 N condition, values in parentheses in the Protrusion column indicate instances where the drill stopped unexpectedly during cutting; these data points were excluded from average, SD, 95 % CI, and *p* value calculation

## Conclusion and discussion

This study proposed a novel dental implant drill system designed to minimize the risk of soft tissue perforation during implant placement. The system incorporated an independently functioning detection mechanism within the drill body, enabling safe bone tissue excavation irrespective of the surgeon’s technical proficiency or patient-specific anatomical variations.

The proposed design is centred on a physical locking mechanism that incorporates a rounded, non-cutting detection tip. Upon the compression of the detector into the drill body, the electrical circuit remained closed, thus enabling the normal rotation of the drill. Conversely, when the detector extended beyond the drill tip, the circuit opened automatically, causing the motor to stop immediately. This mechanism ensured that once the drill passed through the target hard tissue and contacted the underlying soft tissue—represented in this study by the maxillary sinus membrane—the forward extension of the detector triggered cessation of rotation. The rounded, non-cutting tip design ensures that the sinus membrane is not directly subjected to cutting force, thereby preventing unintended tissue damage and further reducing the risk of injury.

In order to assess the feasibility of the system, a prototype drill equipped with the detection mechanism was fabricated, and a perforation experiment was conducted using plywood as a bone model. The drill utilised in the experiment had a diameter of 2 mm, which is consistent with the standard pilot drills employed in clinical dental implant procedures. The findings indicated that when a pressure of 4 N was applied, the mean protrusion was recorded as 0.47 mm. It is important to note that the typical thickness of the maxillary sinus membrane is less than 1 mm. Therefore, the 4 N condition provides a safe margin. The membrane’s inherent elasticity, which has been demonstrated to tolerate deformation of several millimetres during sinus lift procedures, provides an additional degree of safety when drilling is stopped immediately before membrane contact. While the force applied was less than 5 N, an increase to 0.85 mm was observed. The 5 N condition has been demonstrated to present a risk of membrane perforation. It is therefore essential that the pressing force is optimised according to bone density and the specific surgical context, in order to ensure a balance between safety and efficiency.

The present experiment was constrained in its scope, employing a single configuration under dry conditions with constant linear parameters. In order to facilitate more widespread clinical implementation, it is imperative to undertake comprehensive experimental evaluations encompassing a broader spectrum of components. Subsequent studies will employ standardised polyurethane foam blocks, animal bone, and wet conditions to further investigate clinical applicability. Nonetheless, the combinatorial increase in possible configurations presents significant challenges, necessitating substantial preparation time.

In clinical settings, the presence of drilling debris, bodily fluids, and variable surgical techniques poses additional challenges that may compromise the detection mechanism and impair functionality. It is imperative to ascertain whether the proposed drill can maintain performance under such dynamic conditions, given that both cutting load and drill trajectory are subject to non-linear variation due to differences in surgical technique.

It is recommended that future research endeavours entail a more intimate collaboration with clinical experts, with a view to comprehending the actual treatment requirements in the field and refining the drill design. Key investigations will include performance evaluation across varied clinical environments, assessment under non-linear motion trajectories, and comparative studies with different drill specifications. Despite the fact that the present study concentrated on the protection of the maxillary sinus membrane, it is anticipated that the findings will have more extensive applicability to other anatomically sensitive regions, such as the mandibular canal. The objective of these endeavours is twofold: firstly, to substantiate the clinical relevance of the subject under scrutiny, and secondly, to enhance the safety and precision of dental surgical procedures.

## Data Availability

Not applicable.
